# Validation of Acromegaly Quality of Life Questionnaire (AcroQoL) for the Iranian population

**DOI:** 10.1186/s40359-022-00781-0

**Published:** 2022-03-14

**Authors:** Mina Danaei, Leyla Bahadorizadeh, Afsaneh Dehnad, Shirin Mohamadzadeh, Nahid Hashemi-Madani, Mohammad E. Khamseh

**Affiliations:** 1 Social Determinants of Health Research Center, Institute of Futures Studies in Health, Kerman University of Medical Sciences, Kerman, Iran; 2 Antimicrobial Resistance Research Center, Institute of Immunology and Infectious Disease, Iran University of Medical Sciences, Tehran, Iran; 3 School of Health Management and Information Sciences, Iran University of Medical Sciences, Tehran, Iran; 4 Department of English Language, Alzahra University, Tehran, Iran; 5 Endocrine Research Center, Institute of Endocrinology and Metabolism, Iran University of Medical Sciences (IUMS), Firouzeh St, Vali-asr Ave, PO Box 1593748711, Tehran, Iran

**Keywords:** Acromegaly, Quality of life, Disease-specific questionnaire

## Abstract

**Background:**

Acromegaly is a chronic disease significantly affects the physical, emotional, and health-related aspects of patients' life. This study aimed to validate the Acromegaly Quality of Life Questionnaire (AcroQoL) for the Persian-speaking population.

**Methods:**

This cross-sectional study recruited 73 Iranian patients with a confirmed diagnosis of acromegaly. The content validity of the scales was evaluated by an expert panel of eight endocrinologists applying content validity index (CVI) and content validity ratio (CVR). Construct validity was assessed by using confirmatory factor analysis. Internal consistency was assessed on the basis of Cronbach’s alpha, and a goodness-of-fit (GoF) index was calculated to display whether the model fitted the data.

**Results:**

CVI and CVR yielded values of 0.85 and 0.80, respectively (Acceptable CVI: > 0.78 and CVR: > 0.75). The average variances extracted (AVE) from physical and psychological dimensions were 0.520 and 0.462, respectively, exceeding the minimum criterion of 0.40. Cronbach’s alpha for physical and psychological dimensions equaled 0.868 and 0.866, respectively, indicating the adequate internal consistency of multiple items for each construct. The subscales’ R square and path coefficient were greater than the recommended threshold as 0.75 (physical dimension: 0.778, psychological dimension: 0.873), demonstrating the suitability of this criterion. Finally, the GoF value of 0.29 indicated the model's moderate fit.

**Conclusions:**

The findings revealed that the Persian version of AcroQoL is of adequate validity and reliability for evaluating the quality of life of Iranian people with acromegaly.

**Supplementary Information:**

The online version contains supplementary material available at 10.1186/s40359-022-00781-0.

## Introduction

Acromegaly, as a chronic disease, can adversely affect the physical, emotional, and social aspects of patients' life [1, 2]. Acromegaly which is known to be slow in progression and long in duration, occasionally requires life-long medical treatment. It is diagnosed by high level if insulin-like growth factor-1 (IGF-1), adjusted for age and sex, in the presence of non-suppressed growth hormone (GH) of more than 1 mg/dl. This debilitating disease affects patients’ health-related quality of life due to the associated conditions, including cardiovascular complications, cerebrovascular events, gonadal dysfunction, impaired glucose tolerance, diabetes, sleep apnea, impaired respiratory function, and colonic neoplasms. It can also cause emotional distress by affecting the patients' physical appearance [3–5]. Moreover, if the disease persists, the need for life-long medical therapy or radiotherapy may exacerbate the patients' overall health [6, 7].

Several studies have investigated psychological morbidities in patients with acromegaly, indicating that anxiety and insomnia occur in 50% of these patients [1]. General health and appearance are also shown to be particularly and severely affected by acromegaly to scores worse than those of obese patients [2, 8]. It has also been reported that achieving a good biochemical control of the disease is not necessarily associated with a recovery in quality of life [8, 9]. Thus, quality of life should be assessed as an independent outcome in patients with acromegaly, emphasizing the necessity of using an appropriate instrument to accurately assess quality of life in these patients.

Disease-specific questionnaires specifically designed for a particular condition are more likely to assess patients’ self-perceived status and screen patients requiring further evaluation. The Acromegaly Quality of Life Questionnaire (AcroQoL) is the first acromegaly-specific measure originally developed by Badia et al. in 2001 [10]. It consists of 22 items spread across two dimensions: physical (eight items) and psychological (14 items). The psychological domain is further divided into two sub-dimensions evaluating appearance and the diseases' impact on the patients’ personal relationship [10]. The AcroQoL was originally developed and validated for the Spanish-speaking population. Since then, it has been translated into some other languages [11]. It is validated to specifically assess the quality of in patients with acromegaly. This questionnaire has been applied in routine clinics for monitoring patients. AcroQo also has the potential to be used for longitudinal assessment in evaluating the impact of interventions or treatments on the perception of well-being in patients with acromegaly [12, 13].

Additionally, it has been recommended that quality of life be annually evaluated as a principal outcome of acromegaly [14]. According to the World Health Organization (WHO), QoL should be assessed in the context of the culture and values related to the patients’ goals, expectations, standards, and concerns [15]. Thus, the instruments applied for assessment of quality of life should be validated for the specific population for whom they are used. Nevertheless, the Persian version of AcroQoL had not been validated for the Iranian suffering from acromegaly. Therefore, we aimed to validate the Persian version of this questionnaire both for use in the clinical practice and for research purposes.

## Methods

This study was a psychometric research conducted on 73 patients with a confirmed diagnosis of acromegaly attending a tertiary pituitary clinic for their routine follow-up from 2019 to 2021. The patients were included in the study if they were Iranian and had adequate literacy to fill out the questionnaire. They were excluded from the study if they were reluctant to participate in the research and could not fill out the questionnaire.

Acromegaly is a rare disease, and determining the sample size by applying the general principles of sampling (i.e., respondent-to-item ratio) yields an inappropriately high number of participants. It is recommended that, for each question in the questionnaire, at least 2–3 participants be considered. Thus, an appropriate psychometric tool should be employed for the studies with a low-to-moderate sample size in order to have a precise analysis. Thus, by using the smart Partial Least Square (PLS) software, and convenience sampling, the questionnaires were distributed among 75 patients with a confirmed diagnosis of acromegaly; eventually, 73 questionnaires were completed by the participants [16, 17].

Upon obtaining informed consent, we asked the participants to complete the AcroQoL questionnaire. It is a simple questionnaire designed to be self-administered, but for cases where it cannot be self-administered, it can be completed through an interview [11]. The participants completed the questionnaire twice, with a minimum two-week interval. For some participants who had difficulty reading, the questionnaire was read by a researcher and completed through an interview.

The AcroQoL questionnaire has been designed specifically for the evaluation of quality of life in patients with acromegaly. It consists of 22 items spread across two dimensions: physical (eight items) and psychosocial (14 items). The questions are scored on a five-point Likert scale. The responses are categorized as "always, most of the time, sometimes, rarely, never" where the item measures the frequency of the occurrence, and as "completely agree, moderately agree, neither agree nor disagree, moderately disagree, completely disagree" where the item measures the patient’s degree of agreement [11]. Answers are scored from 1 to 5; the response “always” or "completely agree" scores 1, and the response “never” or “completely disagree” scores 5. The higher score the participant achieves, the lower the expected impact of acromegaly on quality of life. The score ranges from 8 to 40 for the physical dimension, and from 14 to 70 for the psychological dimension, and the global score ranges from 22 (worst quality of life) to 110 (best quality of life). To standardize the score for the simplification of interpretation, the items can be scored from 0 (worst quality of life) to 100 (best quality of life) by using the following formula, where Y stands for the recalculated score, and X represents the sum of all the item responses within the dimension or study score (min. is the minimum possible score in the studied dimension, and max. is the maximum possible score in the studied dimension).

$${\text{Y}} = \left[ {\frac{{({\text{X}}) - \min }}{(\max - \min )}} \right] \times 100$$

### Translation

We used the Persian version of the AcroQoL questionnaire translated by the authors who developed the original version of the questionnaire. This questionnaire was originally developed and validated for Spanish-speaking population. Then, it was translated into English and many other languages including Persian using Forward–backward translation procedures. “The Spanish questionnaire was translated by two professional, bilingual translators who were expert in translating health-related quality of life questionnaires; both translations were compared with each other and with the original Spanish version at a consensus meeting; if the translation was clear and correct no changes were made; if there were doubts or contrasting opinions with the project manager, a consensus was reached after in-depth discussion, to produce the first Persian version of the questionnaire. This version was then independently translated back into Spanish to ascertain equivalent significance in both languages. After a second meeting, the second consensus Persian version was produced, and presented to five Persian -speaking patients with acromegaly to asses and correct for comprehension, clarity, cultural relevance and suitable wording (cognitive debriefing), thus providing the final Persian version of the AcroQoL questionnaire” [11].

To quantify the content validity of the AcroQoL, eight expert endocrinologists were asked to examine the necessity/precision of each item for the Iranian culture by using a three-point rating scale (essential, useful but not essential, and not essential) and to rate the items of cultural relevancy, clarity, and simplicity. The content validity ratio (CVR) for every item was calculated by using the formula: [Ne − (N/2)] ÷ (N/2)], where Ne is the number of panelists choosing “essential” for each particular item, and N is the total number of panelists. To calculate the content validity index (CVI), the responses were rated from 1 = not relevant, not simple, and not clear, to 4 = very relevant, very simple, and very clear. Items with a CVI of > 0.78 and CVR of > 0.75 were accepted [18, 19].

### Construct validity

Construct validity is an important type of validity, showing that the instrument measures what it claims to measure. Using confirmatory factor analysis (CFA), we assessed convergent and divergent validity, representative of the construct validity.

### Reliability

Descriptive statistics including frequency, percent, means and standard deviation were calculated for demographic variables. To measure internal consistency and test–retest reliability, Cronbach’s alpha and intraclass correlation coefficient (ICC) were used, respectively. Cronbach’s alpha was categorized as Excellent (α > 0.9), good (0.7 < α < 0.9), acceptable (0.6 < α < 0.7), poor (0.5 < α < 0.6), and unacceptable (α < 0.5). Also, ICC value was categorized as excellent (ICC > 0.90), good (0.7 < ICC < 0.9), moderate (0.5 < ICC < 0.7), and poor (ICC < 0.5) [20].

Having been approved by the Ethics Committee at Iran University of Medical Science (IUMS) (IR.IUMS.REC.1398.526), the study included 73 patients meeting the inclusion criteria.

### Data analysis

Descriptive analysis was applied to analyze the patients’ characteristics. The AcroQol construct was evaluated via CFA in PLS 3. The outer loadings of the measurement indicators (> 0.70) and the average variance extracted (AVE) (> 0.40) of the model’s constructs were examined to establish convergent validity.

Moreover, the Fornell-Larcker criterion was used to demonstrate divergent validity in the cases when the AVE of a composite construct was higher than the construct’s highest squared correlation with any other composite construct [21]. Coefficients of p-value and R square criterion were used to establish the structural model. A greater R square value for the endogenous structural model indicates a better-fitting model. The weak, medium, and strong fitness of the structural model is determined by R square standard and path coefficient. As a rough rule of thumb, R^2^ values of 0.75, 0.50, and 0.25 can be described as substantial, moderate, and weak, respectively. The direction and significance of the path coefficient will determine whether the structural model is fit [22]. A goodness-of-fit (GoF) index was calculated to display whether the model fits the data [23]. SPSS 20 was employed for data analysis, and the results are presented as mean ± SD (standard deviation) and frequency (percentage).

## Results

This study included 73 patients with acromegaly (response rate: 97.33%). The mean (± SD) age of the participants was 42.79 (± 10.53) years, and 54.8% (n = 40) of them were women.

### Content validity

CVI and CVR were applied to assess content validity, which yielded values of 0.85 and 0.80, respectively, representing good content validity.

### Construct validity

**Fig. 1:**
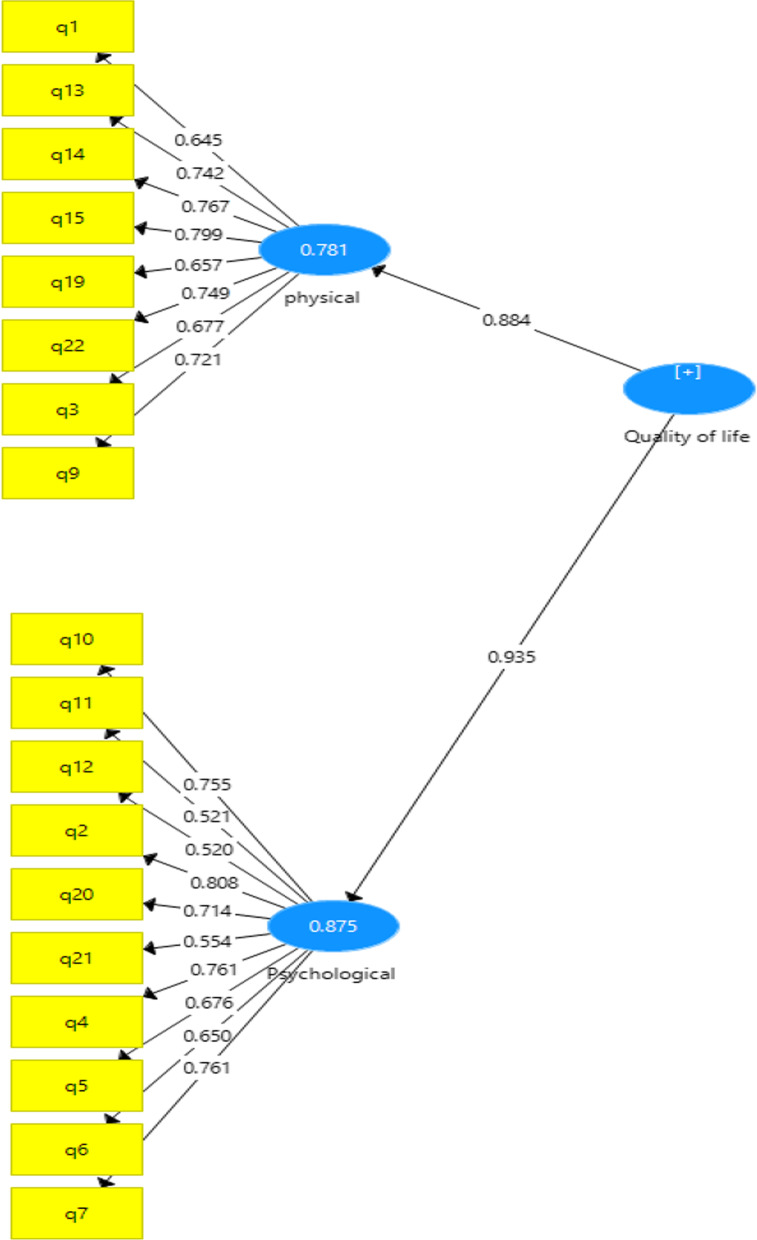
Confirmatory factor analysis of the Persian version of AcroQoL

Convergent validity, tested via CFA, was satisfactory after excluding four questions (questions 8, 16, 17, 18) in that all confirmatory factor loadings exceeded 0.5. The factor loading of all the questions was significant with a range of 0.52 to 0.81 (Fig. 1). In addition, AVE from physical and psychological dimensions was 0.520 and 0.462, respectively, exceeding the minimum criterion of 0.40 [24]. The final instrument included 18 items related to physical (n = 8) and psychological (n = 10) dimensions. The loading factors for the items on each construct were greater than loadings with all the remaining constructs, and the AVE squared of any construct was greater than its correlation values with other constructs (Fornell and Larcker test), suggesting divergent validity (Table 1).

**Table 1:** Validity, structural model, and descriptive results of AcroQoL (Persian version)

Measures	Physical	Psychological
Cronbach’s alpha	0.868	0.866
Composite reliability	0.896	0.894
Average variance extracted (AVE)	0.520	0.462
Physical	0.721	0.679
Psychological	–	0.680
R square	0.778	0.873
Path coefficient	0.866	0.875
T value	34.79	70.82
Significance level	< 0.001	< 0.001
Mean	57.91	65.71
SD	24.64	24.13

### Reliability

Table 1 is shown the distribution of item characteristics. The average total scores of the AcroQoL were (SD:). The sum-scale Cronbach’s alpha was 0.906 that was excellent. Cronbach’s alpha for physical and psychological dimensions equaled 0.868 and 0.866, respectively, indicating the good internal consistency of multiple items for each construct (Table 1). Moreover, corrected item-total correlations, squared multiple correlations, and Cronbach’s alpha if item deleted were calculated. It seems that it is not necessary to delete any item in order to increase internal consistency. Only item 16 had item-total correlations > 0.3.

The ICC was obtained as 0.907 for questionnaire. It was 0.887 and 0.885 for physical and psychological subscales.It was excellent for sum-scale and good for subscales.

### The structural model of the AcroQoL

The subscales’ R square and path coefficient were greater than the recommended threshold (physical dimension: 0.778, psychological dimension: 0.873), suggesting the suitability of this criterion. The t-value derived from bootstrapping in Smart-PLS showed the significance of the effects of variables on one another. All the variables were significant at the confidence level of 0.1%. Table 1 presents a summary of the results of testing the structural model of the AcroQoL. Finally, a GoF value of 0.29 indicates the model's moderate fit.

### Descriptive results

The results of descriptive analyses are demonstrated in Table 1. The mean (SD) of physical and psychosocial dimensions was 57.91 (± 24.64) and 65.71 (± 22.18), respectively.

## Discussion

The results revealed that the AcroQoL questionnaire is a valid and reliable instrument for assessing quality of life in the Iranian population with acromegaly (Additional file 1). The content validity of the Persian version of AcroQoL questionnaire was approved using both qualitative (i.e., inspection of the expert panel members' comments) and quantitative analysis (i.e., a survey of the level of agreement among expert panel members). CVR, indicative of the necessity of the domain’s items, and CVI, representative of simplicity, relevancy, and clarity of the scale’s items, were at the acceptable level [18, 19]. After developing the AcroQoL questionnaire, the developers evaluated its cross-sectional and longitudinal construct validity compared to the other instruments and the General State of Health [25, 26], although CVI and CVR were not evaluated in those studies.

The results of the CFA were promising. AVE from all the constructs exceeded the minimum criterion of 0.40, suggesting that the indicators are more strongly related to their specific construct than to other constructs. Therefore, the divergent validity was verified. However, this was the first study to apply the PLS approach for validation of such a disease-specific questionnaire in patients with acromegaly; thus, comparison with the results of other studies is difficult. However, the validity and reliability of research instruments using PLS has been examined in the previous studies [27]. The translated AcroQol questionnaire demonstrated good internal consistency; the values of Cronbach's alpha for the total score and each subscale were all above the recommended threshold of 0.70, showing high reliability and internal consistency. These results are in line with the findings of the first study reporting the development of the questionnaire. Both the total questionnaire and the two dimensions had a Cronbach's alpha of > 0.80 [10]. Moreover, previous studies demonstrated significant correlations between the entire questionnaire and each dimension of AcroQoL and the generic questionnaires [2]. In addition, test–retest reliability, during a minimum two-week interval, demonstrated good stability, similar to the original version of the questionnaire [11].

The GoF indicators used for establishing the structural model demonstrated values of > 0.75, demonstrating the substantial fit of the model. Finally, the quality of life in this population of patients with acromegaly was in line with the results of previous studies, showing that psychological status has a greater impact on quality of life in this population [28]. Some study examined the quality of life and the associated factors in patients with acromegaly indicated quality of life is reduced in treated patients with acromegaly. Moreover, nadir GH levels and performing radiotherapy associated with the quality of life in these patients [29]. Applying disease-specific instruments to evaluate the quality of life in a specific patient population is of significant advantage as these instruments measure aspects specific to the population in question. Previous studies indicated quality of life is impaired in patients with acromegaly [30, 31]. However, it remains stable after administration of somatostatin analogues [30].

There are isolated reports of the effect of this chronic disease on Iranian patients' quality of life [32]; nevertheless, these studies applied general health-related questionnaires. Thus, the validation of the AcroQoL questionnaire is essential for both the annual assessment of the patients in the clinical practice, and for research purposes.

### Strengths and limitations

This was the first study validating the Persian version of the AcroQoL questionnaire in the Iranian population with acromegaly. The AcroQoL is a comprehensive questionnaire assessing a variety of physical and psychosocial aspects that may affect quality of life in these patients. In addition to the consistent data collection method applied in this study, the participants were recruited from a tertiary center presenting socio-cultural variety, thereby making the results more generalizable to different subgroups of the population. However, this study was limited by the small number of patients and the use of convenience sampling. The simulation shows that the GoF and the GoFrel are not suitable for model validation. However, the GoF can be useful to assess how well a PLS path model can explain different sets of data [33].

## Conclusion

The AcroQol questionnaire, showing to possess appropriate reliability and validity, can be completed in both self-administered and interview-based manners, and is simple to score and interpret. Therefore, it seems to be a suitable instrument for predicting the quality of life in the Persian-speaking population with acromegaly.

## Supplementary Information

**Additional file 1**. Persian version of the questionnaire.

## Data Availability

The datasets generated and/or analyzed during the current study are not publicly available because they are a part of patients’ documents, but are available from the corresponding author on reasonable request.

## Abbreviations

AcroQoL: Acromegaly Quality of Life Questionnaire
AVE: Average variance extracted
CFA: Confirmatory factor analysis
CVI: Content validity index
CVR: Content validity ratio
GoF: Goodness-of-fit
ICC: Intra-class correlation
QoL: Quality of life
