# Supplementary material for: Validation of Acromegaly Quality of Life Questionnaire (AcroQoL) for the Iranian population
Source: BMC Psychol. 2022 Mar 14;10:63. doi: 10.1186/s40359-022-00781-0 (PMC8922868; doi:10.1186/s40359-022-00781-0)
Supplement: Supplementary file 1 — Additional file 1. Persian version of the questionnaire. [file 40359_2022_781_MOESM1_ESM.docx]

**پرسشنامه ACROQOL**

**تاریخ ویزیت:**

**نحوه کامل کردن پرسشنامه**

در ادامه جملاتی را مشاهده می کنید که بیانگر بعضی از مشکلاتی است که بیماری آکرومگالی می تواند در افرادی که مثل شما با این بیماری مواجه هستند، ایجاد کند.

در زیر هر کدام از عبارت های این پرسش نامه چند جواب می بینید. بعضی از این جواب ها مربوط به **تعداد دفعاتی** است که موارد شرح داده شده در عبارات اتفاق می افتند، در حالیکه بقیه جواب ها به **میزان** **موافقت یا عدم موافقت** شما اشاره دارد.

**لطفا جملات را به دقت خوانده، پس از آن گزینه ای را بیش از همه بیانگر حالات شما می باشد با علامت ضربدر مشخص کنید.**

بخاطر داشته باشید که گزینه درست یا نادرست وجود ندارد. ما تنها به آنچه که در حال حاضر به علت آکرومگالی برای شما پیش آمده است توجه داریم.

بسیار مهم است که به تمامی سوالات پاسخ دهید.

**از همکاری شما بسیار سپاسگزاریم**

**به علت آکرومگالی ...**

| 1. **در پاهایم احساس ضعف دارم.** | **4- در عکس ها خودم را ترسناک می بینم.** |
| --- | --- |
| - همیشه 🞏 | - کاملا موافق 🞏 |
| - تقریبا همیشه 🞏 | - خیلی موافق 🞏 |
| - گاهی وقت ها 🞏 | - نه موافقم نه مخالف 🞏 |
| - بندرت 🞏 | - نه زیاد موافق 🞏 |
| - هیچ وقت 🞏 | - مخالف 🞏 |
| 1. **احساس می کنم زشت هستم.** | **5- به خاطر ظاهرم رفت و آمد با دوستان را محدود می کنم.** |
| - کاملا موافق 🞏 | - همیشه 🞏 |
| - خیلی موافق 🞏 | - تقریبا همیشه 🞏 |
| - نه موافقم نه مخالف 🞏 | - گاهی وقت ها 🞏 |
| - بندرت 🞏 | - بندرت 🞏 |
| - مخالف 🞏 | - هیچ وقت 🞏 |
| 1. **احساس افسرده بودن می کنم.** | **6- سعی می کنم از برقراری روابط اجتماعی پرهیز کنم.** |
| - همیشه 🞏 | - همیشه 🞏 |
| - تقریبا همیشه 🞏 | - تقریبا همیشه 🞏 |
| - گاهی وقت ها 🞏 | - گاهی وقت ها 🞏 |
| - بندرت 🞏 | - بندرت 🞏 |
| - هیچ وقت 🞏 | - هیچ وقت 🞏 |

**به علت آکرومگالی ...**

| 1. **خودم را در آینه متفاوت می بینم.** | **10- به علت ظاهرم مردم به من نگاه می کنند.** |
| --- | --- |
| - کاملا موافق 🞏 | - کاملا موافق 🞏 |
| - خیلی موافق 🞏 | - خیلی موافق 🞏 |
| - نه موافقم نه مخالف 🞏 | - نه موافقم نه مخالف 🞏 |
| - نه زیاد موافق 🞏 | - نه زیاد موافق 🞏 |
| - مخالف 🞏 | - مخالف 🞏 |
| 1. **احساس می کنم به خاطر بیماریم مردم مرا طرد می کنند.** | **11- بعضی از اعضای بدن من (مانند بینی، پاها و دست ها) بیش از حد بزرگ هستند.** |
| - کاملا موافق 🞏 | - کاملا موافق 🞏 |
| - خیلی موافق 🞏 | - خیلی موافق 🞏 |
| - نه موافقم نه مخالف 🞏 | - نه موافقم نه مخالف 🞏 |
| - بندرت 🞏 | - بندرت 🞏 |
| - مخالف 🞏 | - مخالف 🞏 |
| 1. **برای انجام امور روزمره ( مثل کار،درس، کارهای خانه،فعالیت های خانوادگی یا فعالیت های تفریحی) مشکل دارم.** | **12- برای انجام بعضی کارها با دستهایم مانند خیاطی یا کار با ابزار دچار مشکل می شوم.** |
| - همیشه 🞏 | - همیشه 🞏 |
| - تقریبا همیشه 🞏 | - تقریبا همیشه 🞏 |
| - گاهی وقت ها 🞏 | - گاهی وقت ها 🞏 |
| - بندرت 🞏 | - بندرت 🞏 |
| - هیچ وقت 🞏 | - هیچ وقت 🞏 |

**به علت آکرومگالی ...**

| 1. **بیماری بر کارایی من در محل کار و یا کارهای روزانه تاثیر می گذارد.** | **16- شب ها خروپف می کنم.** |
| --- | --- |
| - همیشه 🞏 | - همیشه 🞏 |
| - تقریبا همیشه 🞏 | - تقریبا همیشه 🞏 |
| - گاهی وقت ها 🞏 | - گاهی وقت ها 🞏 |
| - بندرت 🞏 | - بندرت 🞏 |
| - هیچ وقت 🞏 | - هیچ وقت 🞏 |
| 1. **در ناحیه مفاصل، احساس درد دارم.** | **17- به خاطر اندازه زبانم، ادای کلمات برای من دشوار است.** |
| - همیشه 🞏 | - همیشه 🞏 |
| - تقریبا همیشه 🞏 | - تقریبا همیشه 🞏 |
| - گاهی وقت ها 🞏 | - گاهی وقت ها 🞏 |
| - بندرت 🞏 | - بندرت 🞏 |
| - هیچ وقت 🞏 | - هیچ وقت 🞏 |
| 1. **احساس خستگی می کنم** | **18- در هنگام رابطه جنسی، با مشکل مواجه هستم.** |
| - همیشه 🞏 | - همیشه 🞏 |
| - تقریبا همیشه 🞏 | - تقریبا همیشه 🞏 |
| - گاهی وقت ها 🞏 | - گاهی وقت ها 🞏 |
| - بندرت 🞏 | - بندرت 🞏 |
| - هیچ وقت 🞏 | - هیچ وقت 🞏 |

**به علت آکرومگالی ...**

| 1. **احساس می کنم آدم بیماری هستم.** | **21- تمایل کمی به رابطه ی جنسی دارم.** |
| --- | --- |
| - کاملا موافق 🞏 | - همیشه 🞏 |
| - خیلی موافق 🞏 | - تقریبا همیشه 🞏 |
| - نه موافقم نه مخالف 🞏 | - گاهی وقت ها 🞏 |
| - بندرت 🞏 | - بندرت 🞏 |
| - مخالف 🞏 | - هیچ وقت 🞏 |
| 1. **تغییرات فیزیکی ناشی از بیماری روی زندگی من تاثیر می گذارد.** | **22- احساس ضعف می کنم.** |
| - کاملا موافق 🞏 | - همیشه 🞏 |
| - خیلی موافق 🞏 | - تقریبا همیشه 🞏 |
| - نه موافقم نه مخالف 🞏 | - گاهی وقت ها 🞏 |
| - بندرت 🞏 | - بندرت 🞏 |
| - مخالف 🞏 | - هیچ وقت 🞏 |

در پایان، لطفا مطمئن شوید که هیچ سوالی را بدون پاسخ نگذاشته اید.

بار دیگر از همکاری شما بسیار تشکر می کنیم.
